# Smart Water Management: An Energetically Autonomous IoT-Based Application for Pressure and Flow Monitoring in Water Distribution Systems

**DOI:** 10.3390/s25237227

**Published:** 2025-11-26

**Authors:** Jonatha B. Silva, Lucas D. de Oliveira, Rafael M. Duarte, Cícero de Rocha Souto, Juan M. M. Villanueva

**Affiliations:** 1 Renewable and Alternatives Energies Center (CEAR), Electrical Engineering Department (DEE), Campus I, Federal University of Paraiba (UFPB), João Pessoa 58051-900, Brazil; jonatha.silva@estudante.cear.ufpb.br (J.B.S.); lucasd.oliveira@estudante.cear.ufpb.br (L.D.d.O.); cicerosouto@cear.ufpb.br (C.d.R.S.); 2 Department of Computer Engineering, Federal University of the São Francisco Valley (Univasf), Petrolina 56304-917, Brazil; rafael.mouraduarte@univasf.edu.br

**Keywords:** artificial neural networks, internet of things, outlier, TEDA, water supply

## Abstract

The distribution of water in urban areas involves several challenges, such as maintaining pipelines, controlling pressure and flow, and monitoring water quality. In particular, the measurement of the flow rate and pressure in pipelines is essential for optimizing water distribution in cities. In recent decades, new technologies have been used to address these challenges, such as hydraulic modeling systems with software, smart sensors, and automated control systems. Among the new possibilities, the use of wireless sensor networks has been highlighted. In this sense, IoT-based nodes have been proposed as a low-cost alternative, with the ability to communicate over the Internet with low energy consumption. Thus, this work describes the necessary steps, challenges, and solutions for the development of an autonomous IoT node applicable to monitoring pressure and flow in a water supply network. In the second part of the work, the data collected by the IoT nodes was processed to eliminate outliers and used to train a model based on artificial neural networks that are capable of predicting the flow in the system under monitoring. The results show that, based on the data measured by the proposed IoT node, it is possible to predict the flow in distribution systems operating in real time.

## 1. Introduction

Water purification and distribution are two basic aspects of life in modern urban centers. Both involve various processes, including the collection, conveyance, treatment, and distribution of potable water to homes. Since its extraction from sources, whether surface or underground, until its use by end consumers and when transported, it passes into water treatment plants, where filtration, coagulation, and chemical correction processes are carried out. After these steps, potable water is stored in reservoirs, where pressure and flow control are required, and, finally, it is distributed to consumers through residential service lines [1].

Accurate measurement of pressure and flow in water distribution systems becomes essential to optimize this process and ensure that consumers receive quality water in the required quantity [2]. With the advancement of research in water distribution, new solutions have emerged, such as the hydraulic modeling software EPANET [3], smart sensors [4], and automated control systems [5]. IoT-based systems and classic wireless sensor networks (WSNs) are increasingly being employed to enhance the efficiency of water distribution systems. Monitoring water distribution systems across urban areas poses implementation challenges. The costs associated with large-scale sensor installation and maintenance can render these systems unfeasible for many municipalities.

There is growing interest in leveraging the capabilities of the Internet of Things (IoT) to develop low-cost energy-efficient solutions: IoT applications enable real-time communication, simple installation, ability of sensors to adapt to changes in the environment, and data collection from a multitude of devices, offering an alternative to traditional sensor networks [6,7].

IoT technology can be used in different scenarios. For example, in the context of healthcare, the Internet of Medical Things (IoMT), i.e., a network of medical devices and applications able to connect to the Internet and communicate with one another, has become more common [8]. IoMT devices collect and transmit real-time medical data to a diagnostic team. To enable healthcare 4.0, new technologies, such as 5G networks, cloud computing, and artificial intelligence technology, are essential.

The combination of continuous monitoring and advanced data analysis has also been used in the management of urban disasters [9,10,11]. Many sensors can be used for IoT implementation to prevent or reduce damage from different types of natural disasters (floods, earthquakes, landslides, and others) [12].

Another common application context for IoT-based systems is precision agriculture [13]. Yan et al. [14] developed a water supply metering system based on the narrow-band IoT (NB-IoT) to use pump energy consumption to indirectly estimate water consumption based on a previously known electricity–water conversion relationship. For this purpose, an AC mutual inductance coil and the energy calculation module are necessary. The measured data is then transmitted to a cloud platform that supports http and https protocols.

Another type of IoT application is the so-called industry 4.0 or IIoT (Industrial Internet of Things). Perhaps one of the largest current issues related to the use of IoT devices is cybersecurity. In water supply systems, cyber attacks could result in the paralysis of the water supply, overflowing tanks, or errors in the quantities of chemicals administered in the water purification process, which could result in a public health calamity [15]. In the present work, cybersecurity was not considered in the development of the proposed solution but was left as a future step.

In order to allow monitoring by human operators and to apply advanced processing and artificial intelligence techniques, the data collected by IoT nodes can be sent to the cloud. In various contexts, this has already been conducted, such as in real-time monitoring of concrete pavement curing compound spraying in the civil construction industry in order to prevent early cracks and distresses, as shown in [16]. Cloud processing of the large volumes of data collected by the IoT has also been harnessed in the context of precision agriculture [17,18]. Another example of this approach is in predicting water levels in urban areas in order to predict flooding by using machine learning and artificial neural networks [19].

In the present work, Wi-Fi in conjunction with the MQTT protocol (Message Queuing Telemetry Transport) and batteries were used instead of LoRa and supercapacitors as they were sufficient to satisfy the project’s needs. The aim is to design and implement an energetically autonomous IoT-based system, powered by a microturbine that generates energy from the flow in the pipe itself, for monitoring pressure and flow in a water supply network. The proposed system combines wireless sensors and low-energy techniques to collect and transmit data in a cost-effective and energy-efficient manner. The proposed approach allows monitoring through a supervisory platform in order to improve the management and optimization of water distribution processes.

In the second stage of this work, data from other quantities collected by the IoT was used to estimate the flow rate of the distribution system. This type of application is known as a soft sensor [20,21]. Indirect estimation of certain quantities through secondary quantities has been suggested in several contexts [22,23,24]. In water treatment systems, an example of a soft sensor application is that created in [25]. In order to estimate oxygen demand (COD), ammonium cation and total nitrogen were measured in wastewater. In water distribution systems, Lima et al. [26] used soft sensors to estimate water flow. The incorporation of the detection and treatment of outliers and the analysis of uncertainties by means of Monte Carlo dropout in the indirect estimation of flow in water supply systems was carried out by Alencar et al. [27], managing to improve the quality of the estimate.

In summary, the main contribution in the present work is the development of an IoT and cloud-based water flow soft sensor system for distribution plants. The energetically autonomous IoT-based platform for monitoring pressure and flow can be controlled remotely and is powered by power harvesting assisted by micro-hydroelectric turbines, which are moved by the water that passes through the pipe that is being monitored. The forecast is conducted using artificial neural networks and includes a procedure for detecting and compensating for outliers.

After this Introduction, some works related to IoT-based applications, water storing, purification, and distribution systems are shown in Section 2. The technical details regarding the main building blocks of the proposed monitoring system (microturbine, sensors, etc.) are shown in Section 3. The interconnection of the ESP32/Wi-Fi module pair with the voltage regulator was completed with a printed circuit board whose details are provided in Section 4. The methods used to reduce the energy consumption of the proposed IoT system are described in Section 5. Moving on to the development stage of the forecasting system, Section 6 discusses outliers and the detection and treatment methods. In Section 7, details about the ANN model used for forecast flux and its project are shown. The results obtained are divided into two types: the cost and energy consumption characteristics of the proposed system and an example of forecasting operation. Both are set out in Section 8. Finally, the main conclusions drawn from the work and the possibilities for future research are set out in Section 9.

## 2. Related Works

When it comes to water supply systems, IoT nodes can also be developed to monitor water quality and consumption, as in [28]. In that work, the proposed system consists of three elements: an end-user node (a microcontroller SX1276, sensors, batteries, and its management system), data storage unity (cloud server and storage system), and a means of connecting to the Internet (local gateway). The node’s power source is outdoor solar cells. The communication and network protocol chosen was LoRa due to its great range and low energy consumption, and the MQTT protocol was chosen to enable communication with the server. The cloud-based server is used to store and generate data regarding water quality and consumption available to users.

The use of IoT-based devices of the batteryless type, that is, with energy storage elements alternative to batteries, has been proposed, with the use of algorithms that make efficient use of stored energy, as proposed in [29]. Two modules with LoRa transceivers SX1276 and ESP32 combined were fed by supercapacitors that store energy generated by solar cells. Supercapacitors have a higher cost per kWh, which increases the cost of the system. In the present work, we want to present a low-cost IoT device, which makes this storage undesirable.

In order to avoid residential tank overflows and the resulting waste of energy, an IoT-GSM-based device was proposed in [30]. This device can be controlled remotely via the app. Furthermore, the water supply can be interrupted via the app if the water shows signs of contamination. Using IoT-based solutions is indicated as cost-effective and affordable for residential buildings. An ultrasonic sensor is used to check the availability of water in the tank, while a flow sensor is used to determine the existence of losses before the supply line based on previously calibrated values. A turbidity sensor is used to monitor water quality. The communication is based on GSM modules, and the user can receive an SMS or an e-mail about the system’s status.

An IoT device using the MQTT protocol was proposed in [31] for monitoring water tank quality, level, and temperature. In this system, temperature, level, leak, and quality sensors are used. The MQTT protocol is used in the fog layer, and the server sends messages to appropriate users (consumers, counselor, or cooperation office). Just like the present work, cloud technology was used in that work. The functioning of the proposed device was verified through tests under different conditions (contaminated water, altered pH, leaks, etc.) and the results were displayed on a status dashboard. At the edge layer, an Arduino is used in conjunction with a Wi-Fi module to communicate with the area gateway. In the present work, microcontroller ESP32 is used in the edge layer.

In [32], the authors developed bioinspired optimization algorithms to assist in the scheduling of pumping stations based on parameters informed by the IoT. The schedule plan specifies the quantity of water to be provided to each region. These actions can be carried out automatically without assistance from a human hand based on data obtained with the help of IoT smart sensor nodes that monitor the water distribution structure both in accessible and remote places. The idea is to reduce energy costs through careful choices regarding pump start and discharge times. Such choices would be made by using bioinspired algorithms. The genetic algorithm (GA), Particle Swarm Optimization (PSO), and Binary Dragonfly Algorithm (BDA) approaches were used in this work. Among them, BDA was the algorithm that most reduced water losses: 27% against 23% with PSO and 15% with GA.

In [33], the authors implemented a prototype IoT system for monitoring water consumption in a specific region of Spain. The proposed network is based on LPWAN (Low-Power Wide Area Network) communication protocols and is capable of performing real-time measurements in order to reduce costs and increase the performance of the water supply network. As in the present work, ultrasonic sensors were used to perform measurements per unit hour, compared to measurements performed twice a month with traditional systems. The data collected can be monitored with the help of an online platform called SMARTWATER. The results were compared with those obtained using the traditional system. The new system was able to identify 382 incidents of failure, compared to 22 for the traditional system. This allows the organization’s reaction time to be shorter and resource management to be more efficient.

Ebisi et al. [34] stated that the integrated use of the IoT and AI provides the most promising strategies for detecting water leaks in infrastructure on an industrial scale and in smart cities. The authors applied artificial intelligence techniques to detect leaks in distribution systems.

Saritha et al. [35] proposed an IoT-based mechanism for monitoring of water quality. The authors demonstrated that the use of the IoT is capable of monitoring real-time PH and water temperature. Garg et al. [36] carried out monitoring water quality through the use of the IoT and machine learning. The authors applied a mechanism for monitoring water properties and predicting whether the water is safe for consumption. The authors achieved an accuracy of 96.12% in detecting the water quality.

Boebel et al. [37] proposed a monitoring system for the water distribution infrastructure in which energy is obtained from the temperature difference between the soil and the pipeline using a thermoelectric generator (TEG). In that case, data transmission is carried out through an LoRaWAN network. Their proposed system is capable of generating 21 Joules per day, which would ensure sensor data transmission on 94% of days.

Another water IoT monitoring system was proposed by Syrmos et al. [38], which is subdivided into five subsystems: (1) flowmeter, (2) water quality unit, (3) LoraWAN modules, (4) cloud infrastructure, and (5) machine learning models. Their flowmeter was based on an STM32L073XX Arm Cortex MCU, chosen because of its energy consumption and compatibility with LoraWAN. The water quality unit includes a pH sensor, an oxidation–reduction potential sensor, an electrical conductivity sensor, and a temperature sensor. To deal with the data generated by so many sensors, cloud infrastructure was used. As was correctly pointed out by the authors, cloud hosting provides high computing power, which benefits data storage and ML training. In that paper, machine learning was used to produce estimates of future water quality and consumption such that anomalies associated with leaks could be detected. Qualitative and quantitative estimations were performed using two ANN-based machine learning models. In that research, the IoT system was installed in homes, so the use of power harvesting was not necessary.

In Puviyarasi et al. [39], a monitoring strategy of water quality was presented. To achieve this, the authors used an ESP32 microcontroller for data acquisition and transmission to a remote server, Farebase. In the approach developed, the authors used sensors to measure the turbidity, temperature, and pH of the water. The developed system demonstrated efficiency in monitoring water quality in different scenarios, alerting operators whenever the quality values exceeded the thresholds. The hardware used was designed to be low-cost, consuming little energy. However, the authors did not make predictions with the acquired data. The alert generated in monitoring consisted only of indicating that the values acquired are outside the parameters configured in the proposed system.

Aragonés, Oliver and Ferrer [40] proposed a batteryless narrow-band IIoT to monitor petroleum refinery. The purpose of this IoT is to measure the vibration of electrical machines in order to predict the occurrence of failures. It harvests residual energy from industrial surfaces through the Seebeck effect. Field tests were successfully carried out on a hot water pump. The system they proposed is another one that uses power harvesting to provide the IoT node energy autonomy.

Many works cited throughout this section have in common the application of IoT devices for the efficient management of water supply systems. Some have technological choices in common with the present work, such as cloud storage and the use of the ESP32 microcontroller and the MQTT protocol. A notable difference in the system proposed here is the fact that the focus is on the design of the IoT node, with an emphasis on power harvesting over the water flow in the pipe itself, providing advantages such as autonomy in energy management for a long period of time, the ability to communicate with cloud platforms, and low cost. Some of the works cited also combine the use of advanced processing techniques and artificial intelligence with data collection using IoT devices. This work proposes, in addition to developing the IoT, the development of a complete soft sensor using the data collected to predict future flow in water distribution systems. Through the use of outlier detection with TEDA, and subsequent correction, the proposed soft sensor is capable of making more accurate predictions.

## 3. Proposed Monitoring System

Before going into the details of its main components, the behavior of the system will be described in a general way.

**Figure 1:**
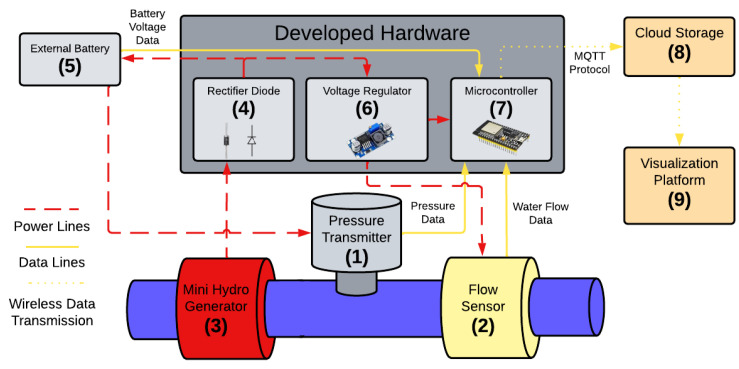
Proposed monitoring system. Source: authors.

In this work, the proposed monitoring system consists of a set of hardware and software technologies, as shown in Figure 1. The main components for system’s operation are (1) a pressure transmitter, (2) a flow sensor, and (3) a small hydroelectric generator or microturbine. This generator is connected to (4) a redundant rectifier diode, which acts in a complementary way to the microturbine rectifier bridge when charging (5) the external battery. It is essential for maintaining system’s stability, managing the charging process of the battery. The laboratory conditions for carrying out the experiments were as follows: ambient temperature (27 ± 2 °C), water supply from the LENHS pilot system pressurized by a centrifugal pump, and the use of 2-inch PVC pipes with an outlet reduced to a half-inch diameter, with the inlet flow controlled by a frequency inverter.

The external battery is the energy reservoir of this system, which supplies power to the system’s components. The energy storage guarantees continuous system operation when the water flow is too low and the microturbine might not provide sufficient power. This power harvesting mechanism, based on water flow, is the basis of this system’s energetic autonomy.

The diode model used was the IN4007 and is considered redundant because, as will be explained later, the microturbine has its own encapsulated rectifier. In the proposed circuit, the diode works as an additional way to prevent the reverse flow of current to the microturbine.

The sixth component is (6) a voltage regulator that serves to adjust the voltage values supplied to the (7) microcontroller to safe levels (7). This microcontroller collects, processes, and transmits via the Internet to (8) a cloud storage system and (9) visualization platform to visualize pressure, flow, and battery voltage level data. Monitoring the battery level is important to prevent it from exceeding operating limits.

**Figure 2:**
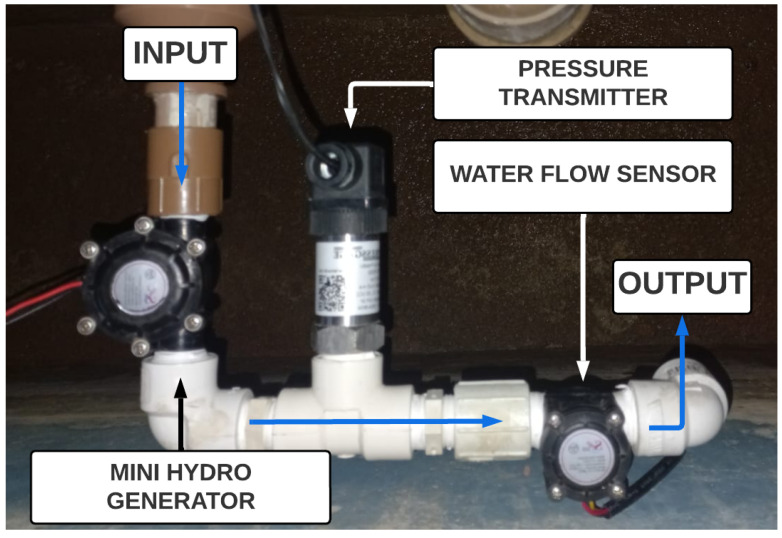
Experimental setup. Source: authors.

The experimental setup is illustrated in Figure 2. The operation of the proposed monitoring system was evaluated using half-inch pipes. It is important to mention that the installation order of each component was not randomly defined. In this setup, the first component is the microturbine, which was placed before the right-angle curve because its location experiences large pressure loss. The pressure transmitter was positioned right after the angle curve because it is the heaviest element in the assembly; it can result in excessive torque. At the end of the pipe, at another right-angle curve, the flow sensor was positioned. In other words, the positioning of each component was mainly due to mechanical reasons.

### 3.1. Industrial Pressure Transmitter TPI-PRESS

This pressure transmitter was chosen due to its high-quality transmitter, precision, and durability. Its construction in a stainless material allows its use in a wide variety of applications. Its measurement range is from 0 to 40 mH_2_O (3.92 bar), with a current response from 4 to 20 mA.

### 3.2. Flowmeter YF-S201

This flowmeter is based on Hall effect and is composed of an internal rotor housed by an ABS plastic structure. The rotor is turned by the passage of water. Its speed depends on the rate of flow, which is related to a pulse signal in the output. The microcontroller then reads the pulses and, based on the amount, outputs speed information. YF-S201 is able to measure flows between 1.5 and 30 L per minute.

### 3.3. Microturbine

**Figure 3:**
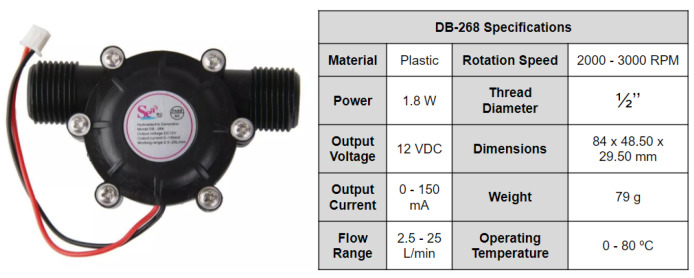
Mini hydroelectric generator. Source: authors.

The microturbine, also known as a micro-hydro or pico-hydro generator, is illustrated in Figure 3. It is a device that converts the energy of small falls and currents of water in electric energy. It is a renewable source useful for power harvesting systems in hard-to-reach areas, where access to electricity may be limited, as is the case in this research.

The operation principle behind a mini hydroelectric generator is the conversion of potential energy at higher elevations in electric power. This is the same reason why most hydroelectric power plants are located near waterfalls or dams, and why the microturbine has to be located just before the right-angle curve.

A three-phase generator that incorporates a design where magnetized teeth are housed inside the microturbine is the element that realizes the energy conversion. The flow of water over the generator causes these teeth to rotate, which changes magnetic field. This changing in magnetic field induces an electrical current in the coils present within these teeth, according to Faraday’s law of electromagnetic induction [41].

**Figure 4:**
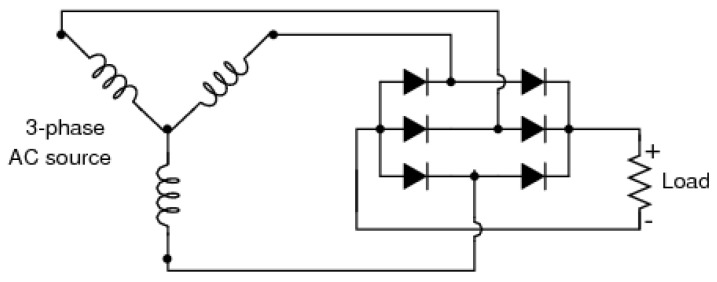
Mini hydroelectric generator electrical model. Source: authors.

The electrical current generated is an alternating current (AC). However, many systems, including the proposed system, require a direct current (DC). That is why the generator output circuit includes 6 diodes, 2 for each phase (illustrated in Figure 4), which serve as complete-wave three-phase rectifiers. Thus, there will only be current flow in one direction.

To stabilize the output voltage, the generator also includes a 12 V voltage regulator that receives as input an imperfect DC voltage, which can vary due to changes in the flow rate of water and other factors, and outputs a constant 12 V DC. Associated with the voltage regulator, the design includes an output capacitor to further smooth out the output voltage and reduce ripple. The capacitor stores electrical charge and releases it when the voltage drops, acting like a temporary energy storage, keeping the voltage level more steady.

In this research, a model DB-268 plastic housing microturbine was used for meeting the necessary specifications for the development of the system.

### 3.4. Battery

Battery specifications were chosen as 12 V and 7 Ah, meaning it can deliver a current of 7 A for an hour, with 12 V in its terminals before needing to be recharged. The model chosen was a battery for nobreak.

### 3.5. Voltage Regulator LM2596

This high-efficiency step-down regulator (voltage reduction) is capable of converting voltages from input ranges between 4.5 V and 40 V at adjustable output voltages from 1.25 V to 37 V and has a high conversion efficiency, which can reach up to 92%.

### 3.6. Microcontroller ESP32

Developed by the company Espressif, the ESP32 is a microcontroller that presents a module of Wi-Fi and Bluetooth communication, a system with dual-core processor. In the project, it was chosen due to its low price compared to microcontrollers on the market with Wi-Fi communication modules.

### 3.7. Internet of Things

In the last decade, Internet of Things rapidly became integrated into various applications. The Internet of Things (IoT) is a system of interconnected computing devices with unique identifiers (UIDs) that can transfer data across a network autonomously, i.e., without any kind of human-to-human or human-to-machine interaction. In the context of industrial instrumentation, IoT can be applied by connecting sensors and microcontrollers to the Internet through the use of a machine-to-machine (M2M) communication protocol.

With the development and popularization of wireless communication technologies, there is growing interest in the development of new applications of IoT in daily life, providing new amenities and services for users based on communication possibilities between objects or between objects and users. With new possibilities come new challenges, such as enhancing safety, protection, comfort, communication, technical management, and autonomy.

In industrial systems, supervisory and monitoring systems are becoming more interconnected. Consequently, IoT solutions are cost-effective, requiring low power consumption and straightforward installation and configuration [42].

In addition to the need for monitoring in the management of water resources, the use of IoT becomes important due to its data generation capacity, which is necessary for the development of environmental planning studies and water resource management.

**Figure 5:**
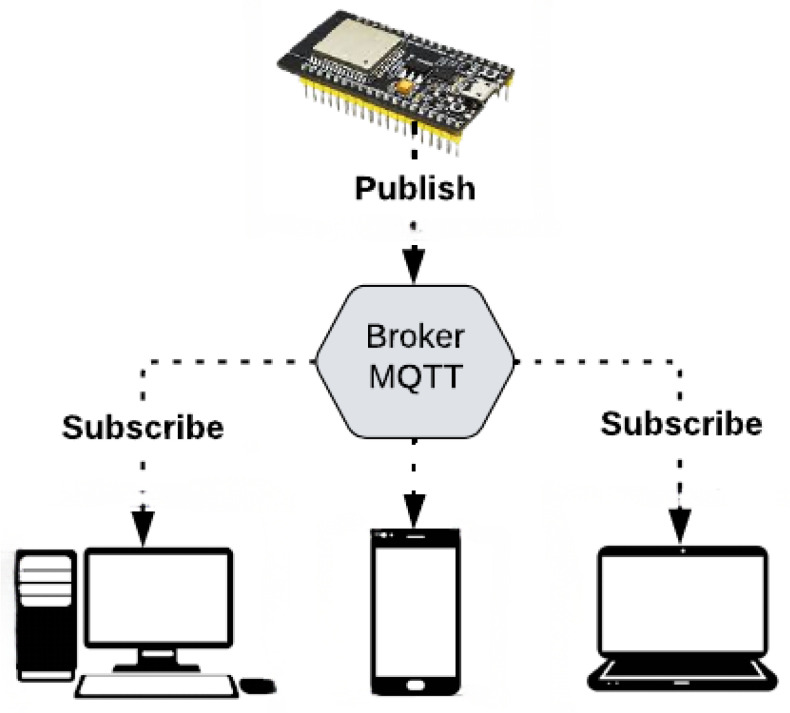
Operation of the MQTT protocol. Source: authors.

After ensuring that the local measurement system is working correctly, the next step is to provide the data to a platform with Internet access. In this research, the MQTT (Message Queuing Telemetry Transport) communication protocol was chosen due to its simplicity and excellent performance. This protocol is based on a Publish–Subscribe model (illustrated in Figure 5), in which each client communicates with a central broker, forming a many-to-many connection; i.e., the broker manages and distributes messages from one or more subscribers to one or more subscribers. In this scenario, the role of clients is to connect to the broker, publishing messages and subscribing to receive messages from other clients. In other words, clients never directly connect to other clients, instead relying on the broker for message handling.

In the proposed system, the broker was established in a hosted manner, using the MQTT library for the ESP32 microcontroller. The main reasons for this choice are the low cost and the need for low energy of the modules with built-in Wi-Fi and Bluetooth.

## 4. Developed Hardware

In this section, the hardware development is presented in detail, ranging from the conceptualized printed circuit board to the planned encapsulation.

**Figure 6:**
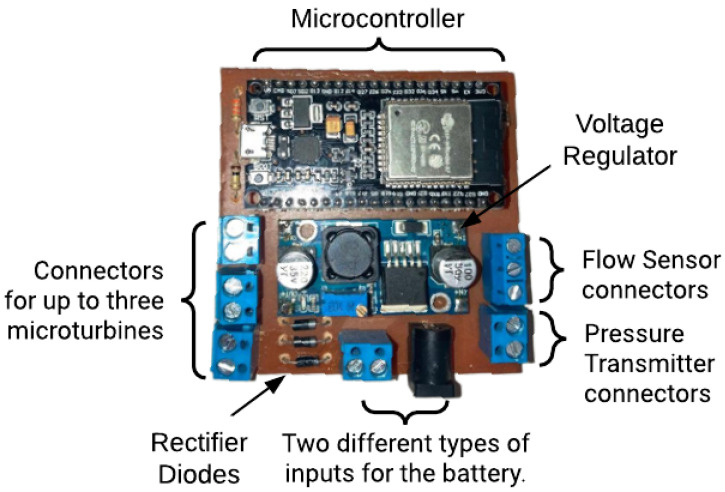
Developed hardware. Source: authors.

After testing the circuits in a breadboard, it was possible to move to the development stage of the printed circuit board for the proposed hardware, as illustrated in Figure 6. This rigid and flat board holds two elements of the proposed system. These are as follows:

- **Microcontroller ESP32**: low-cost microcontroller associated with a Wi-Fi communication module, a dual-core processor system, hybrid Bluetooth, and multiple built-in sensors. This module makes the construction of IoT systems simpler and more compact.
- **Voltage Regulator LM2596**: a buck (or step-down) voltage regulator, capable of converting input voltages between 4.5 V and 40 V into adjustable output voltages ranging from 1.25 V to 37 V. The main reason for its choice is its high conversion efficiency, which can reach up to 92%. In addition, the device includes protection features against overload, short-circuit, and overheating, which guarantees safe and reliable operation.

To save energy, only passive components, that is, elements that absorb, store, or dissipate electrical energy, were used in some parts of the system. This is the case for circuits used to read the battery voltage and water pressure, consisting of two simple resistive dividers. It is important to highlight that it is not necessary for the flow sensor. The proposed hardware supports the simultaneous use of up to 3 microturbines. For this reason, the board is equipped with 3 redundant rectifier diodes. Additionally, a P4 connector has been utilized to enable the use of both batteries and common commercial power sources when the connection to a local electrical grid is possible.

**Figure 7:**
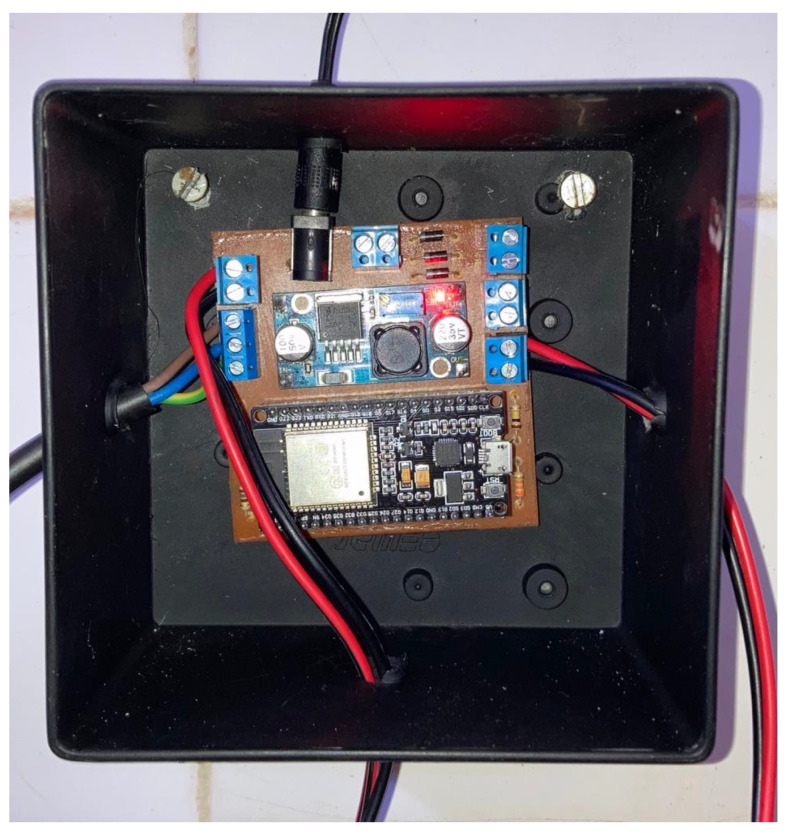
Prototype encapsulation. Source: authors.

The encapsulation of the prototype was carried out using a commercial model of plastic box. It was adapted to accommodate the printed circuit board as well as the necessary conductors for sensing and powering through custom-made perforations. Due to the high humidity in the probable facility, it is highly recommended to have the maximum sealing of the external environment in addition to using covers. The inside of the box is shown in Figure 7.

## 5. Energy-Saving Techniques

To achieve the desired energetic autonomy of the monitoring, it is necessary to use energy-saving tools. Thus, in addition to the prior choice of low-consumption components, some techniques were employed.

### 5.1. DeepSleep Technique

The DeepSleep mode of the ESP32 allows the microcontroller to enter a low-power state, with the purpose of saving battery energy and extending the device’s lifespan [43]. When in this mode, the processor and most of the ESP32 components are turned off to minimize power consumption. This feature leads the ESP32 to consume as little as 10 μA, which enables extending the device operation time, even with a low-capacity battery. However, to use this feature, it is necessary to program the microcontroller to enter the low-power mode at the appropriate time and set the events that should wake up the device. Additionally, it may be necessary to reconfigure the device peripherals and temporarily interrupt processing to ensure the lowest possible energy consumption. For the system proposed in this work, a reading is taken per minute. In other words, the microcontroller “sleeps” for 1 min and then “wakes up” and takes a reading.

### 5.2. Storage and Transmission of Data Packet

**Figure 8:**
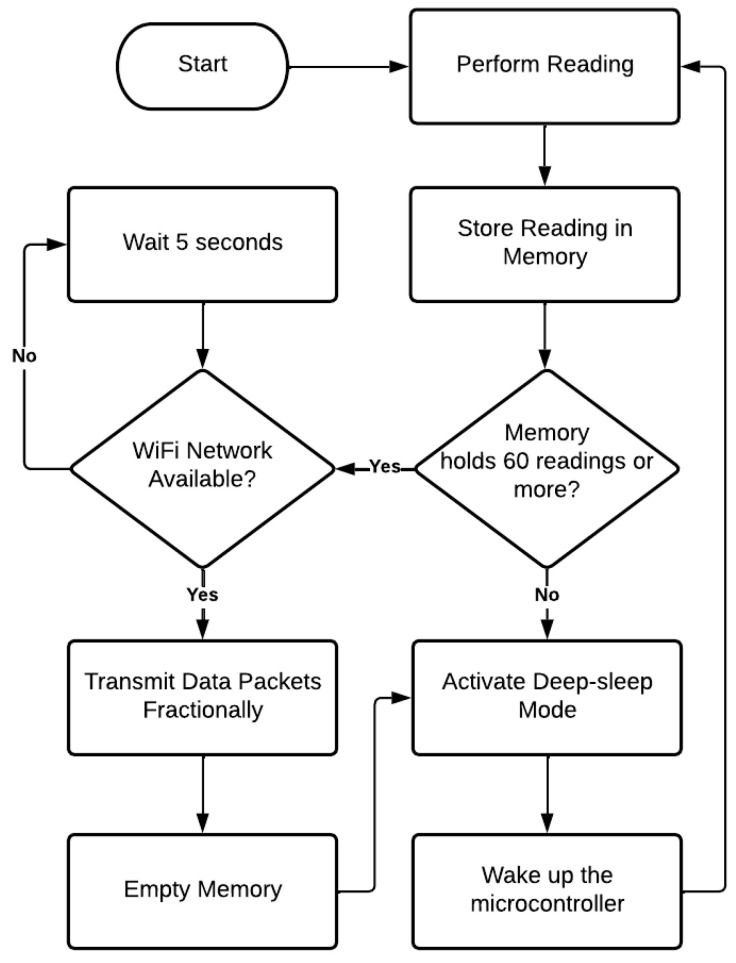
Flow diagram representing microcontroller’s operation. Source: authors.

Although the reading is carried out minute by minute, data transmission to the application layer every minute would mean activating the microcontroller’s Wi-Fi module 60 times an hour. This approach would be power-intensive due to the ESP32 Wi-Fi module’s constant connection and transmission requirements. Thus, it was chosen to bundle 60 readings of each of the three variables into one packet per hour. If an SD card module were used for storage, power load would grow even more. Thus, it was decided to explore the ESP32’s RTC memory as an alternative. This memory is non-volatile and offers 8 KB storage capacity, making it a proper solution for this project. This entire process is summarized by the flow diagram in Figure 8.

## 6. Outlier Detection Techniques

Failures in the reading of monitoring sensors may be present in a set of data, which are considered errors, which, if not treated or ignored, can compromise trend analysis [44]. In the study carried out by Figueiredo et al. [45], the authors excluded data that presented inconsistencies, such as negative values, or data deviation in the comparison with reference parameters or historical information from a water company from Brazil, leaving explicit the presence of outliers in the measured data. TEDA is preferable to MAD (Median Absolute Deviation) filtering [46] in continuous (online) datastreams because it allows for the detection of outliers in an adaptive real-time manner, calculating the typicality and eccentricity of each incoming point without needing to store the entire history, unlike MAD, which is better suited for static data. That is why it was chosen for this work.

### 6.1. Typicality and Eccentricity Data Analytics—TEDA

A data analysis framework based on typicality and eccentricity was introduced by Angelov [47]. TEDA is a technique that requires low-effort computation, inserted in the data analysis repertoire, aimed mainly at outlier detection.

Initially proposed by Angelov [47], the technique aims to identify anomalies in datastreams. The algorithm is based on two fundamental concepts: typicality and eccentricity. Typicality represents the similarity of a sample with the rest of the sample dataset, while eccentricity expresses the differences regarding the sample in relation to the same set.

This technique, as it is simple to implement, can be implemented in several platforms, including microcontrollers, with the main focus being application in data flows. Another advantage of this algorithm is its ability to be used without prior training, and it can be used both online and offline. From the moment the data is received, the algorithm evolves with the classification, requiring low computational effort and making it a viable option for embedded systems.

When considering the datastream as a vector, the sum of the Euclidean distances for each of the elements is calculated. Equation (1) presents the sum of the distances from a specific sample (*x*) to each of the other *k* elements.

(1) $$\pi_{k}\left(x\right)=\sum_{i=1}^{k}d\left(x,x_{i}\right).$$

In this way, the eccentricity of the data sample *x*, at time instant *k*, can be given by

(2) $$\xi_{k}\left(x\right)=\frac{2\pi_{k}\left(k\right)}{\sum_{i=1}^{k}\pi_{k}\left(x_{i}\right)},\;\mathrm{para}\;k>2,\;\sum_{i=1}^{k}\pi_{k}\left(x_{i}\right)>0,$$

The equation can be simplified so that the eccentricity can be calculated recursively, not needing to calculate all distances for each new sample. Otherwise, it would need to store all samples in memory to update the calculation of the distance of each sample in relation to the others. To calculate eccentricity, it is necessary to calculate the mean and variance of the set of samples received.

The mean of *x* at time *k* is given by Equation (3), which uses the previous mean calculated. For the first sample, the mean is considered to be the first value

(3) $$\mu_{k}\left(x\right)=\frac{2\pi_{k}\left(k\right)}{\sum_{i=1}^{k}\pi_{k}\left(x_{i}\right)},\;\mathrm{para}\;k>2,\;\sum_{i=1}^{k}\pi_{k}\left(x_{i}\right)>0,$$

The eccentricity can be calculated recursively. Otherwise, you would need to store all samples in memory to update the calculation of the distance of each sample in relation to the others. To calculate eccentricity, it is necessary to use the mean and variance of the set of received samples.

The mean of *x* at time *k* is given by Equation (4), which uses the previous mean calculated. For the first sample, the mean is considered to be the first received value.

(4) $$\mu_{k}=\frac{k-1}{k}\mu_{k-1}+\frac{1}{k}x_{k},\;\mathrm{where}\;\mu_{1}=x_{1}.$$

The variance is calculated by applying Equation (5). The variance takes the value equal to zero for the first data read.

(5) $$\sigma_{k}^{2}=\sum_{i=1}^{k}\frac{{\left(x_{i}-\mu_{k}\right)}^{T}\left(x_{i}-\mu_{k}\right)}{k}.$$

The eccentricity at time *k* is given by

(6) $$\sigma_{k}^{2}=\frac{1}{k}+\frac{{\left(\mu_{k}-x_{k}\right)}^{T}\left(\mu_{k}-x_{k}\right)}{k\sigma_{k}^{2}}.$$

It is observed that, to calculate the eccentricity, it is enough to have the average values and variance of the dataset, which in the computational application are updated recursively for each new received set of data. Typicality can be calculated as the complement of eccentricity. After calculation, the eccentricity is normalized, ensuring that the sum of all terms of eccentricity is equal to 1.

Other common methods use the standard deviation of samples to calculate outliers; however, this approach is restricted to Gaussian distributions [48]. Differently, TEDA offers another approach for calculating outliers, not requiring the sample distribution to resemble the normal distribution.

Using the Chebyshev inequality, Equation (7), in which the probability of a sample is less than or equal to $1/m^{2}$, where *X* is the input data, μ is the mean, σ is the deviation default, and *m* is a positive value greater than 1:

(7) $$P\left(||X-\mu ||\geq m\sigma \right)\leq 1/m^{2}.$$

Based on the work of Bernieri, Betta and Liguori [49], Angelov adapted the use of normalized eccentricity for the expression defined in Equation (8), which can be used to characterize whether or not a piece of data is an outlier.

(8) $$\zeta >\frac{m^{2}+1}{2k}$$

Once anomalous data is detected, it can be deleted or replaced with other data using a data correction technique.

### 6.2. Outlier Treatment

The outliers detected have to be replaced using interpolation techniques to fill in missing data methods. Some methods include Linear Regression, Support Vector Machine (SVM), and replacement of the gap by the average of the adjacent data. In this work, in order to analyze the offline data, the anomalous data was replaced by interpolating the previous data. For this purpose, polynomial interpolation using the Lagrange method is used [50].

For the online application, if the data is identified as an outlier, linear extrapolation is used, in which

(9) $$y_{n+1}=y_{n}+\left(y_{n}-y_{n-1}\right)$$

In this way, it is possible to replace the outlier identified by extrapolating the $y_{n+1}$ value based on the last two known values $y_{n}$ and $y_{n-1}$.

### 6.3. Applying TEDA Offline

With the data measured in the laboratory, Google Collaboratory was used to access the MySQL server hosted on AWS Amazon. Initially, the Amazon AWS server is accessed via the mysql.connector library. The data read represents the emulation of the water consumption of a household over the course of a day.

With the data stored in a dataframe, four columns are evident: time, pressure, flow, and voltage, with sixty values stored in each. As mentioned, this set of values represents the emulation of a household’s consumption. The time data was modified so that the sixty equally spaced values were distributed between the times of a full day, starting at 00:00 and ending at 23:59. This was completed by disregarding the seconds so that the flow rate was read every 24 min.

## 7. Forecast Model

An artificial neural network is composed of the input layer, hidden layer, and output layer. Its training takes place by adjusting the synaptic weights between artificial neurons [51].

Mathematically, a neural cell can be simulated from adding or subtracting the input signals and applying an activation function, which removes the linearity of the operation and enables the network to learn and represent more sophisticated patterns, transforming the linear combination of signals into an output that can capture the complexity of the behavior of the system under analysis.

The neural network is optimized by adjusting the cost function, which can be optimized by gradient descent. From balancing the synaptic weights, it is possible to configure the network to acquire the desired response. Optimizers, like the descending gradient, are used to make it easier to find the global minimum in the cost function, but with implementations that aim to converge more quickly [52]. Specifically, the Adam optimizer has an adaptive learning rate [53]. This means that different parts of the neural network can have different learning rates, which is difficult to control manually. This feature speeds up the convergence of the cost function and has high performance compared to other optimizers. That is why we chose it.

### 7.1. Data Collection for the ANN Project

**Figure 9:**
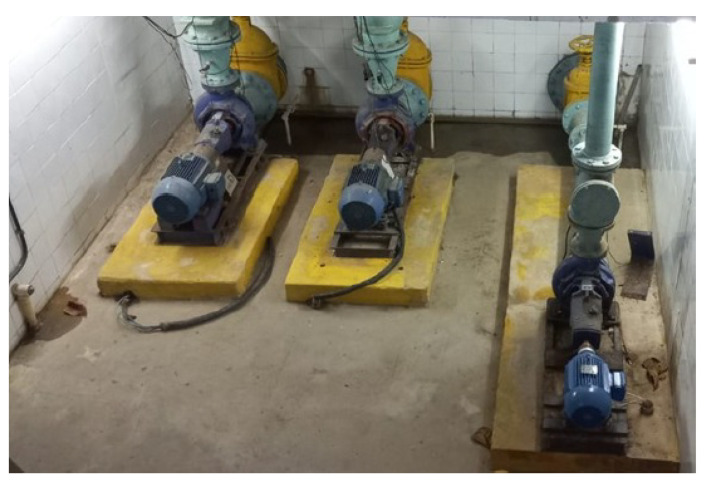
Experimental setup used to collect data used in ANN training.

The laboratory used in this research has a network of pipes pressurized by a centrifugal pump coupled to a three-phase induction motor, which pressurizes a network of pipes that simulates a water distribution system. A frequency inverter is used to control the system, allowing the rotation of the motor–pump combination to be controlled. Figure 9 shows a representation of this system.

By modifying the inverter frequency via SCADA BR, it is possible to change the pressure and flow rate at the LENHS measurement point. In view of this, five variables were selected for input to the prediction model: inverter frequency and current and previous pressure and flow measurements.

Once the flow and pressure data had been properly grouped, delayed inputs were created, corresponding to the previous flow data, which consisted of the ANN input variable. It was also found that the estimated flow rates show a strong correlation with the inverter frequency and the measured pressure and flow values.

### 7.2. Artificial Neural Network Project

**Figure 10:**
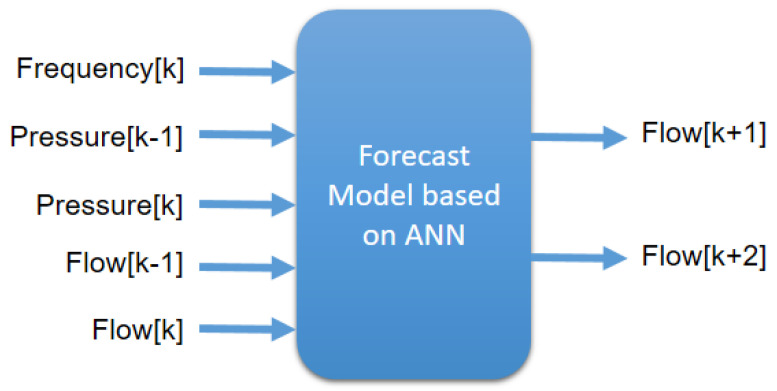
Artificial neural network with five inputs and two outputs. Source: authors.

Figure 10 shows a diagram of the input and output data of the artificial neural network. In this figure, *k* corresponds to the current instant, while k+1 and k+2 represent future forecasts. Similarly, k−1 indicates the samples lagged by one sampling period.

The proposed standardization procedure is a mandatory requirement for re-adapting the data so that the neural network can be trained. In order to improve the network’s response, the input data was normalized by dividing it by the maximum expected value.

The data was divided into training (72%) and testing (28%). In order to make the number of samples more flexible for future work, the algorithm was developed to carry out this proportion regardless of the amount of data, thus adjusting the data to the established proportion.

The training data was used to train the neural network, which has delayed inputs of measured flow and pressure. The input data consists of the value of the flow and pressure measured on the experimental bench, as well as the input value of the inverter responsible for pressurizing the system. As output, the two future flow rates to be measured by the system are predicted.

During network training, in order to verify the best response, a number of activation functions were analyzed: Relu, Sigmoid, Tanh, and Softmax. The neural network was trained for each activation function. The error of the ANN was stored at the end of each iteration in a dictionary. During the process of selecting the activation function, a value was chosen for the number of epochs that did not compromise processing time.

After training the network, test data was applied to predict future flows, and the error presented by the ANN was observed, as well as graphically examining the flows and pressures read at the experimental bench.

### 7.3. Forecast Test

**Figure 11:**
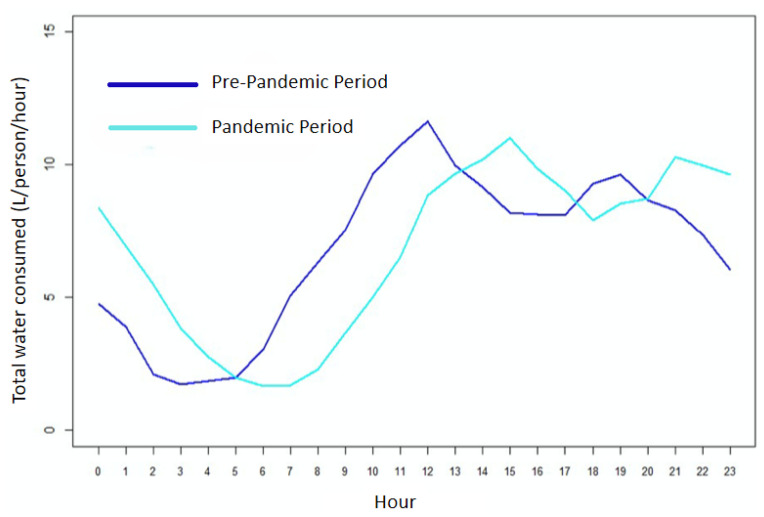
Hourly water consumption profile for the pre-pandemic and pandemic periods. Source: adapted from Cominato et al. [54].

In this work, in order to carry out the tests, a water consumption profile reported in the literature by Cominato et al. [54] was adopted. That profile was emulated using the frequency inverter. The graphical response of the relationship between the inverter input data in the supervisory system and the response of the flow rate read in the monitored pipe is shown in Figure 11. The inverter frequency was varied from 30.0 Hz to 54.91 Hz in a set of 60 defined values.

## 8. Results

In this section, we will first show how the monitoring system works and its costs. Its analysis is divided into three distinct areas: (1) advances achieved in the areas of current consumption, (2) software, and (3) hardware and costs involved. After exploring how the IoT works, the performance of the soft sensor in flow prediction will be analyzed with and without treatment of outliers. The results obtained using data previously stored in a database and results with real-time measurements will be shown.

### 8.1. Energy Consumption

In tests carried out in the absence of water and with different constant water flow rates, an analysis of the current drawn from the battery was carried out in different scenarios. These results are shown in Figure 11.

From these results, the preliminary success of this application can be seen. This graph represents the behavior of current drained from the battery for different water flow rates measured in the piping. Each flow value corresponds to four bars: in blue is the current drained from the battery when the microcontroller is in DeepSleep state, orange represents the current drained in operating mode, and green shows the maximum current drawn from the battery when the microcontroller starts transmitting data via Wi-Fi, and, in red, there is a weighted average current. In a timespan of one hour, the average approaches the value of the DeepSleep current since the microcontroller spends 96.5% of the time in DeepSleep, 3.33% in reading mode, and the remainder transmitting data to the cloud.

As the flow rate was increased, the drained current decreased due to the generation of a mini hydroelectric generator until, at a flow rate of 14.85 L/min, a negative current began to appear; that is, the system was no longer draining current from the battery but providing battery current. This behavior is increased even more when the water flow increases. Observing the profile curve, it can be seen that the flow rate remains above 16 L/min for approximately 14.4 h, during which more than 9798 J (=(12.6 V) · (15 mA) · (14.4 h) · (60 min) · (60 s)), or 2.7 Wh, produced by the microturbine and not consumed by the IoT node circuits, are supplied to the battery. On the other hand, the battery provides about 5560 J (=(12.6 V) · (15 mA) · (14.4 h) · (60 min) · (60 s)), or 1.54 Wh, for 6.4 h, where the flow rate is around 11 L/min. Outside these ranges, the flow rate remains mostly above 14.8 L/min, as can be seen in Figure 11, so there would be no consumption of energy stored in the battery. In this way, energy autonomy is achieved

**Figure 12:**
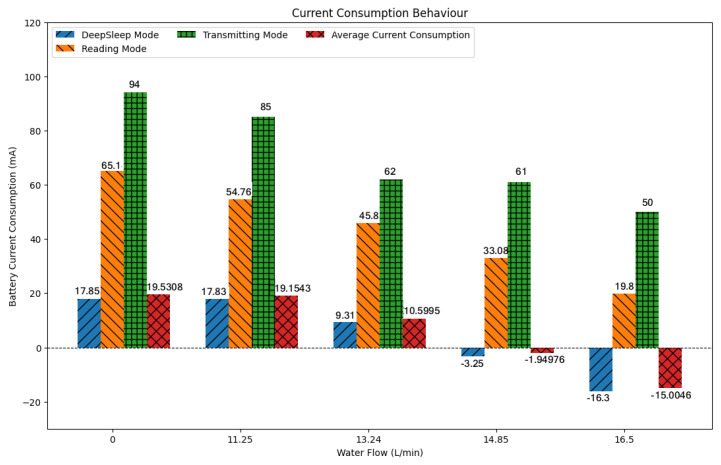
System’s consumption profile. Source: authors.

From Figure 12, it is clear how using DeepSleep mode while the ESP32 is not transmitting is what guarantees the energy surplus in the battery as the average current ends up approaching the current in DeepSleep mode.

This result reinforces the idea that adding new mini hydroelectric generators in series would reduce the flow necessary to reach the state of energetic autonomy, in addition to increasing the timespan that the batteries will stay charged.

### 8.2. Software

**Figure 13:**
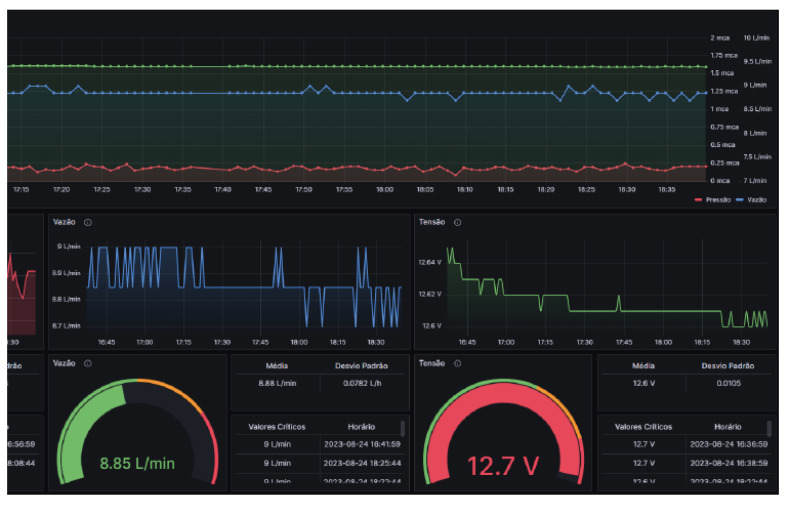
Grafana platform being used as a supervisor. Source: authors.

The monitoring system works based on sampling several physical variables related to the state of the water pipe, as shown in Figure 13. In addition to displaying the readings of each variable and the time when it was sampled, the average, maximum value, minimum value, and standard deviation of each variable are also calculated. To facilitate data visualization, Grafana was used as a data supervisory system.

### 8.3. Hardware and Costs

**Figure 14:**
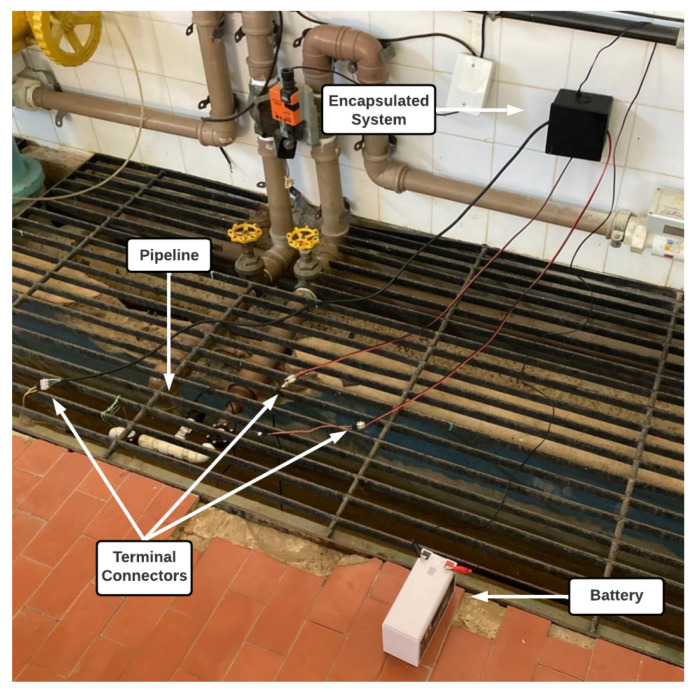
Monitoring system installation in the pipeline. Source: authors.

In Figure 14, the physical installation of the proposed system in the pipeline to be monitored is shown, in addition to the 12 V 7 Ah battery (a smaller-capacity battery could be used and placed inside the encapsulation).

In Table 1, the cost of the proposed system is calculated through the costs of its main components. Developed in 2023, the total cost of the IoT was USD 70.68.

### 8.4. IoT Measurement and TEDA Results

**Figure 15:**
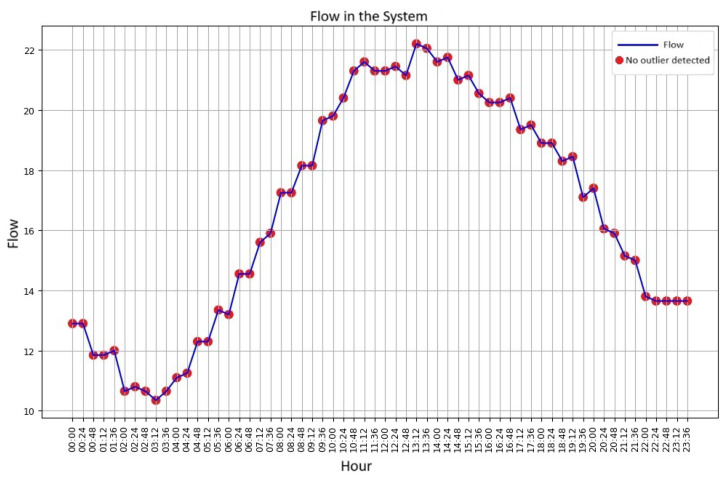
Flow profile generated in the laboratory. Source: authors.

The graph of the flow values collected by the IoT system, from the emulation in the laboratory’s hydraulic plant, similar to the average water consumption in a household over the course of a day, is presented in Figure 15.

**Figure 16:**
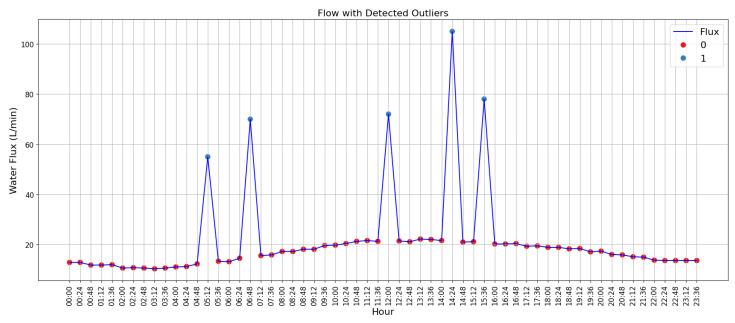
Data acquired from LENHS, with detection of five outliers. Source: authors.

However, the data acquired may contain reading errors or missing data values, which could compromise the interpretation of the distribution system’s behavior. In order to correct these anomalies, the algorithm proposed by Angelov [55] was applied, with which it is possible to identify the presence of outliers in the data received. To test the algorithm, random values were assigned to five data records to simulate failures in reading the flow sensor, thus generating outliers in the set of data read. Figure 16 shows the dataset with the outliers identified after applying TEDA.

**Figure 17:**
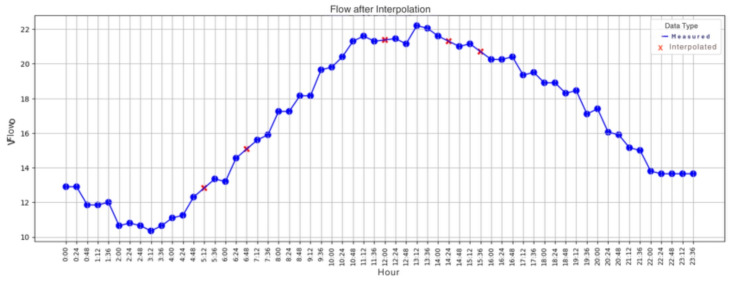
Data acquired in the laboratory, with outliers corrected. Source: authors.

After identifying the error in the dataset, linear interpolation was applied, which consists of the average between the samples before and after the sample detected as an outlier, thus being used to replace the data identified by the algorithm as an outlier. Figure 17 shows the new graph after correcting the data identified as heterogeneous by TEDA. The relative error of replacing the actual value with the interpolated value (both expressed in L/min), for samples whose indices are indicated, is shown in Table 2.

This process of detecting and correcting anomalies in the data is fundamental to this work because the presence of an outlier would generate errors in the application of the artificial neural network used to predict future flows.

In this work, TEDA was also used to emulate the system running online. Flow, pressure, and inverter frequency values were acquired from the network in order to simulate a stream of sequential data and thus be able to examine TEDA’s behavior in detecting outliers.

The data used for the online TEDA study was acquired from the same plant used to generate the data for the offline study (referenciar a figura do supervisório). To carry out the outlier detection analysis, a specific script was developed with the aim of randomly inserting outliers into the dataset. Inserting outliers consisted of adding a random number between −100 and 100 to the data read, thus generating an anomaly in the measured data. The percentage of outliers inserted was 3%, guaranteeing a controlled number of anomalies, which allows for a more realistic analysis of the system. This process of generating outliers was applied to each of the records in the dataset, allowing the inclusion of controlled anomalies.

The TEDA algorithm was then applied to the modified set to check its ability to detect outliers. This procedure was repeated several times, varying the proportion of outliers in the dataset in order to assess how TEDA behaves under different conditions of noise in the data.

TEDA’s performance was analyzed using the accuracy calculation, which is a performance metric that indicates the proportion of correct predictions in relation to the total number of predictions. *Accuracy* ranges from 0 (no correct predictions) to 1 (all correct predictions), representing the fraction of total correct predictions. *Accuracy* can be obtained by applying

(10) $$Accuracy=\frac{TP+TN}{TP+TN+FP+FN},$$

in which *TP* is the number of true positives, *TN* is the number of true negatives, *FP* is the number of false positives, and *FN* is the number of false negatives.

**Figure 18:**
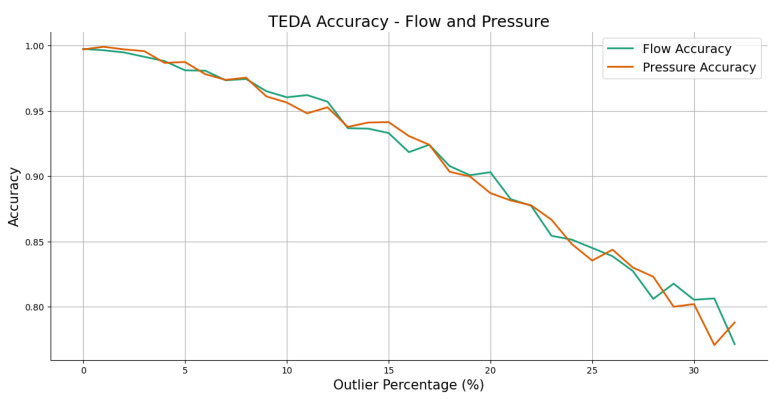
Accuracy of TEDA in the variation of the percentage of outliers inserted in the flow and pressure readings. Source: authors.

**Figure 19:**
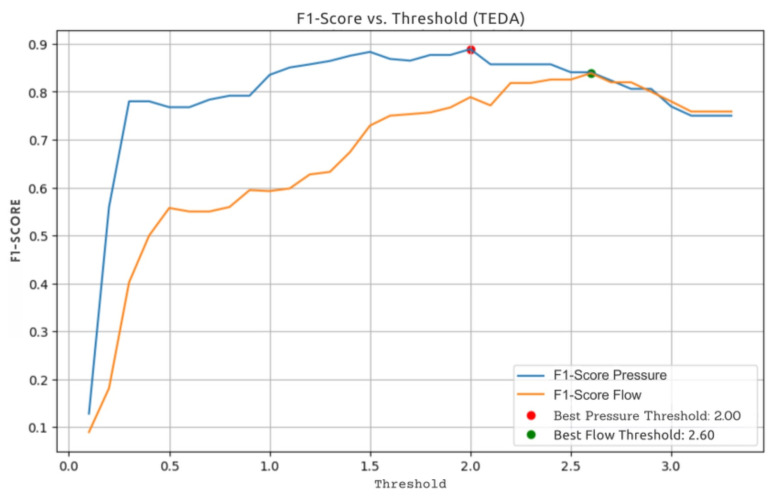
Variation of TEDA’s F1-score in adjusting outlier detection sensitivity. Source: authors.

Figure 18 shows the results obtained from the iterations carried out, demonstrating the accuracy of the TEDA application as a function of the percentage of outliers present in the dataset. Figure 19 shows that TEDA has an accuracy of over 95% for a percentage of outliers of up to 10%. When there are more than 30% outliers in the dataset, TEDA is less than 80% accurate.

The procedure was repeated using the same dataset but this time varying the value of the threshold *m* in the TEDA algorithm. The aim of this stage was to assess how different values of *m* influence TEDA’s accuracy in detecting outliers. For each value of *m*, TEDA was applied to the same dataset, making it possible to observe variations in the algorithm’s performance in terms of accuracy and ability to correctly identify outliers. This process made it possible to choose a value of m that had the best accuracy for the data analyzed, guaranteeing more efficient and consistent outlier detection.

To measure TEDA’s performance in detecting outliers in pressure and flow measurements, the F1-score was used, according to Equation (11). The F1-score is a performance metric that represents the balance between accuracy and sensitivity in a classification model. In summary, the F1-score was used because it is more reliable than accuracy in problems with imbalanced classes, such as outlier detection.

(11) $$Accuracy=\frac{\frac{TP}{TP+FP}\times \frac{TP}{TP+FN}}{\frac{TP}{TP+FP}+\frac{TP}{TP+FN}},$$

Figure 19 shows the results obtained with the different threshold variations.

### 8.5. Application of ANN for Flow Forecasting

After carrying out the stage of detecting errors in the read data, the next step is to train and apply an artificial neural network to identify future flows in the distribution system.

To train the MLP–ANN (multilayer perceptron artificial neural network), the inverter frequency was used as one of the parameters of the neural network’s input layer. The other parameters used were the flow and pressure measured by the ESP-32. In summary, the neural network configured has five neurons in the input layer and two neurons in the output layer. The ANN follows the model proposed in Section 7 for flow prediction.

The aim is to use the inverter frequency, pressure, and flow of the system at different times to estimate the flow forecast for two intervals of data readings ahead, in this case corresponding to the system’s response in 48 future minutes, consisting of two 24-min samples.

**Figure 20:**
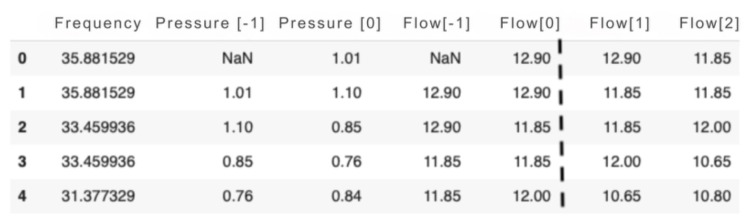
ANN input data. Source: authors.

The dataframe adjusted for the training input of the network is shown in Figure 20. The first five columns represent the data that feeds the input layer. The last two columns, corresponding to the future flow reading, are used in the output neurons for supervised training of the neural network.

**Figure 21:**
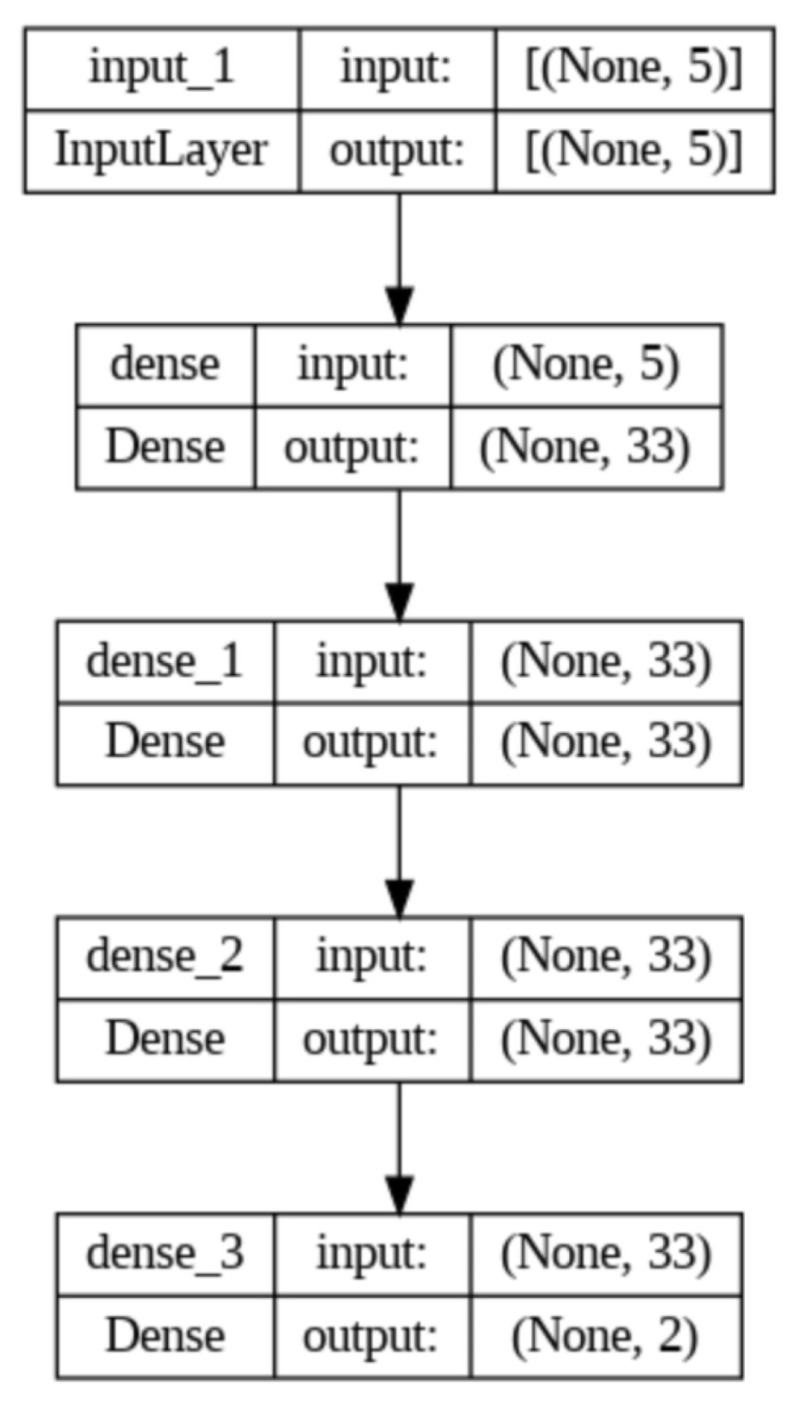
Artificial neural network used. Source: authors.

In addition to the input layers, three hidden layers with 33 neurons each were used, as shown in Figure 21. This choice was made using the grid search method, in which various configurations were tested and the one with the best performance was selected.

To train the network, the data was divided into 72% for the training set and 28% for testing. To define the activation function of the neurons, the network was trained with 300 epochs for the following activation functions: hyperbolic tangent, Sigmoid, Relu, and Softmax. At the end of each training session, the mean squared error is calculated and the function with the lowest error is used for ANN training with 700 epochs.

After the final training, the network is used to predict the flow of two readings ahead. It can be seen that the output of the neural network is normalized as a result of the normalization process applied to the data before training. This procedure is essential to ensure that the input and output variables are on the same scale, preventing values with very different magnitudes from unduly influencing network’s learning. In order to restore the values to their original scale and thus recover the real amplitude of the flow readings, a denormalization process was implemented. This process consisted of multiplying the eigenvector resulting from the network by the respective eigenvalues used in the normalization stage. In this way, it was possible to reverse the effects of normalization and obtain the real output values.

As results, the ANN showed an MSE of $4.4284\times 10^{-5}$, which reflects good performance since this value indicates a minimal difference between the network’s predictions and the actual data. In addition, the MAPE error was 3.15%, which means that the neural network predictions showed an average deviation of only 3.15% from the observed values. The MAPE is within the expected estimation range, with a value below 5%.

The low MAPE value stems from the network’s ability to cope with data variability, keeping predictions within an acceptable error range for real-time applications such as monitoring supply networks. These results are especially important for sanitation systems in the Brazilian context, where efficiency in water resource management is crucial for dealing with challenges such as water scarcity and the optimization of distribution networks.

### 8.6. Use of ANN Trained with Outliers: Evaluation Without Application of TEDA

**Figure 22:**
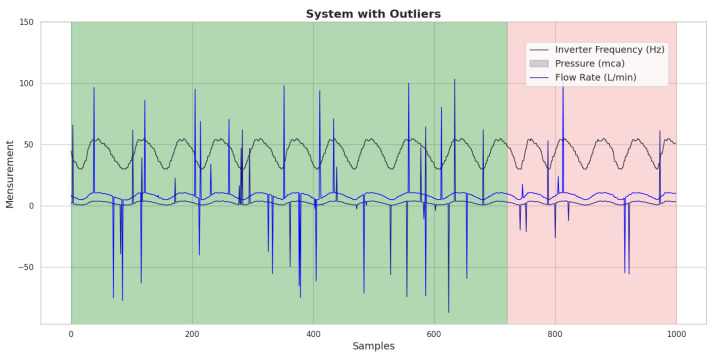
Graph with the consumption forecast, considering the presence of outliers generated in 3% of the data. Source: authors.

In order to identify the impact of the presence of outliers on the training and application of the ANN, an algorithm was applied to generate outliers at a rate of 3%, randomly, in the training and testing datasets. The introduction of these outliers is intended to simulate real conditions in which atypical data can affect the network’s performance. The resulting dataset, after inserting the outliers, can be seen graphically in Figure 22, where the green background indicates the data used to train the ANN and the pink indicates the data used for testing. This visualization allows for a clearer analysis of changes in data patterns and their potential effect on the ANN’s ability to learn and generalize from the altered dataset.

**Figure 23:**
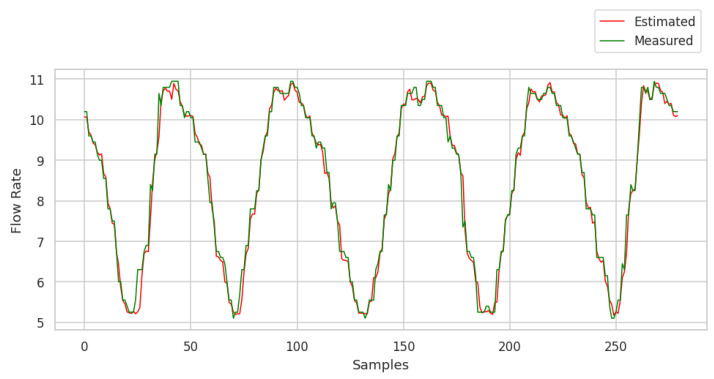
Graph with the consumption forecast with the presence of outliers generated at a rate of 3%. Source: authors.

After training the neural network and applying the test data, the graphical response to the consumption demand forecast can be seen in Figure 23.

The results obtained from the application of the ANN with the presence of outliers during training and in the test data are shown in the graph displaying the prediction values. The ANN showed a mean square error (loss) of $1.6716\times 10^{-5}$, indicating robust performance in modeling consumption demand forecasts, even in the presence of atypical data. This loss value suggests that the network’s forecasts are very close to the real values, demonstrating the ANN’s ability to deal with the variability introduced by outliers. In addition, the MAPE recorded was 3.4%. This result indicates that, on average, the network’s predictions deviated by approximately 3.4% from the real values. This level of accuracy is acceptable for practical applications, highlighting the effectiveness of ANN in non-ideal conditions.

### 8.7. Use of ANN Trained Without Outliers: Performance Evaluation on Test Data with Outliers Without the Application of TEDA

The unpredictability of outliers is a significant challenge in ANN training. Although a network can be trained under conditions in which the sensors are operating correctly, the presence of various external factors, such as sensor faults, environmental interference, or operational anomalies, can result in errors in the readings that are not detected during the training phase. These outliers can compromise the network’s ability to generalize and predict accurately in real situations.

**Figure 24:**
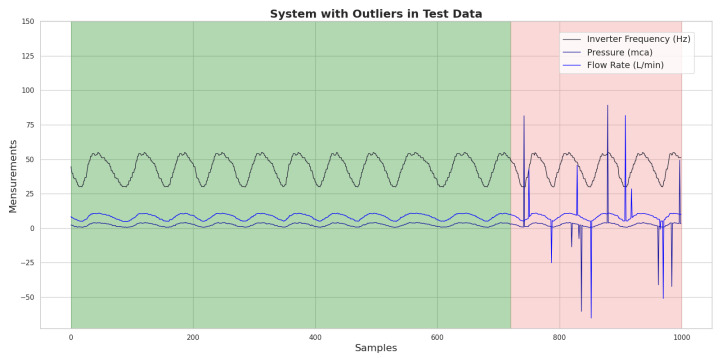
Graph of the dataset with the presence of outliers generated at a rate of 3% only in the test data. Source: authors.

To address this issue, a training strategy was created in which the network was fed a dataset free of outliers, ensuring that its learning was based on consistent and reliable information. However, in order to assess the RNA’s resilience to atypical data, outliers were deliberately inserted, at a rate of 3%, only into the test data. This approach, shown in Figure 24, where the green background indicates the data used to train the ANN and in pink the data used for testing, allows us to observe how the network behaves when confronted with predictions in a scenario that reflects the unpredictability of real-world conditions.

Through this methodology, it is possible to analyze the RNA’s ability to detect and deal with outliers that may occur in practical situations while ensuring that the initial training is not jeopardized by inconsistent data. This analysis is crucial to understanding the robustness of the model and its effectiveness in forecasting demand in monitoring and control systems, where the accuracy of readings is fundamental to making informed decisions regarding forecasts in a scenario that reflects the unpredictability of real-world conditions.

The results obtained with the application of the ANN, when the outliers were inserted only in the test data, are shown graphically in Figure 24. This approach made it possible to evaluate the network’s performance in a scenario that simulates the presence of reading errors that can occur in the real world.

The data showed that, in this context, the ANN presented a mean square error (loss) of $3.8067\times 10^{-4}$. This value indicates a significant increase in error compared to previous situations in which the network was trained without outliers. This result suggests that the presence of atypical data in the test data negatively impacted the ANN’s ability to accurately predict consumption demand, reflecting the difficulty the network faces when dealing with information that does not align with the data pattern it was trained on.

In addition, the mean absolute percentage error (MAPE) obtained was 5.16%, indicating that the ANN predictions showed an average deviation of approximately 5.16% in relation to the real values. Although this level of error is acceptable in many contexts, it represents a deterioration in accuracy compared to the previous performance, when the ANN managed to maintain an MAPE of 3.4%.

These results highlight the importance of considering the unpredictability of outliers when developing predictive models. The ANN’s ability to maintain its accuracy when trained with consistent data is an advantage, but the introduction of outliers in the test data highlights the model’s vulnerability to unforeseen errors. Therefore, analyzing performance in scenarios with outliers is fundamental to understanding how to improve the robustness of the network and ensure that it can adapt to variable conditions, more adequately reflecting the complexity of real monitoring and control systems.

In this context, the application of the TEDA method to detect the presence of outliers is of paramount importance. Early detection of atypical data can enable adjustments to be made to the training process and, consequently, improve the accuracy of ANN predictions. This scenario is addressed in the next section, which presents how TEDA can be implemented to optimize the network’s performance in the face of data with outliers.

### 8.8. Using ANN Trained Without Outliers: Performance Evaluation on Test Data with Outliers Using TEDA

**Figure 25:**
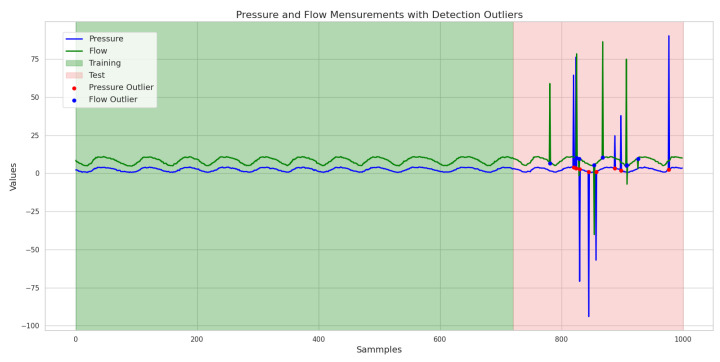
Graph of TEDA applied to detect outliers at a rate of 3% on test data only. Source: authors.

After training the ANN with and without outliers, as well as applying the network to test data containing outliers, we can now observe the detection of these anomalies. Before applying the test data with outliers, the TEDA method was implemented to identify the anomalies present in the dataset. The graphical response illustrating the detection of outliers by TEDA is shown in Figure 25, which provides a clear view of the distribution of the data and the points considered to be atypical.

**Figure 26:**
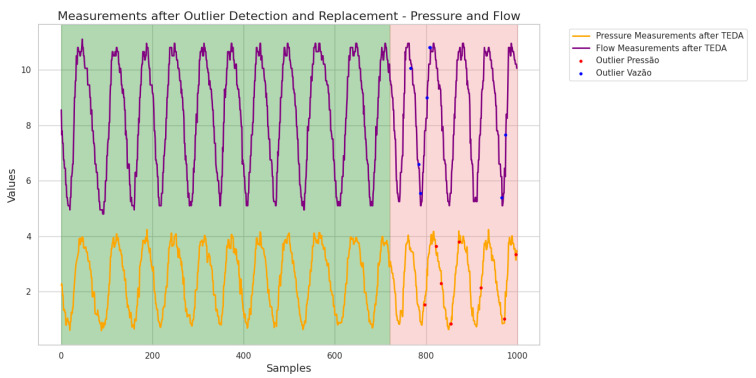
Outliers replaced after TEDA detection. Source: authors.

After detecting the outliers using the TEDA method, the data identified as atypical was removed from the dataset. To ensure the continuity and integrity of the information, these values were replaced by the last reading recorded before the anomalies occurred. This approach aims to minimize the impact of anomalies on the ANN’s predictions and improve the quality of the dataset for future analysis. The graphical response of the new dataset, after this replacement, can be seen in Figure 26, allowing a clear comparison between the original and adjusted data. In this figure, the green-highlighted region corresponds to the data segments that were corrected and subsequently used for both training and testing of the ANN, ensuring consistency and continuity in the input sequence. The pink-highlighted region, on the other hand, indicates the portion of the corrected dataset that was exclusively reserved for testing, allowing the evaluation of the model’s generalization capability under conditions that replicate real operational scenarios while avoiding bias from training exposure.

**Figure 27:**
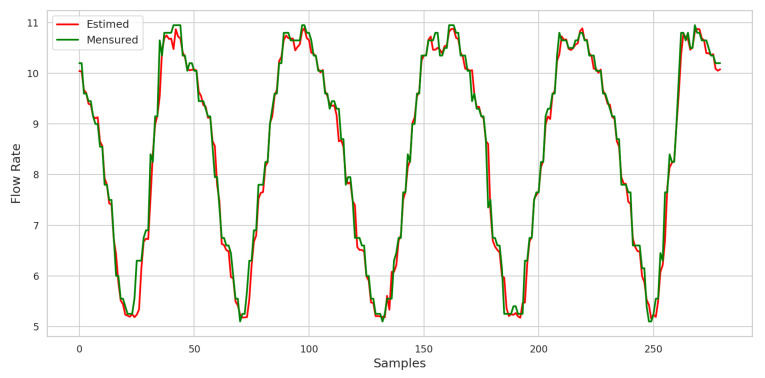
Waveform obtained with RNA application after using TEDA. Source: authors.

The results obtained by applying the ANN after removing the outliers and replacing them with the latest recorded values are shown graphically in Figure 27. This step was crucial in assessing the effectiveness of the ANN in predicting consumption demand in a cleaner dataset free of anomalies.

With the implementation of TEDA and the corrections made, the ANN showed a mean square error (loss) of $4.4329\times 10^{-5}$. This value indicates a significant improvement in the network’s performance compared to previous tests, suggesting that the ANN can now better capture the patterns in the data. In addition, the MAPE obtained was 3.04%, reflecting an average deviation of only 3.04% from the real values. This level of accuracy is indicative of the effectiveness of the outlier detection and treatment process, showing that the interventions carried out have contributed to a more robust network performance.

These results reinforce the importance of detecting and treating outliers when building more reliable predictive models, showing that the application of TEDA was a fundamental step in optimizing the performance of the ANN.

### 8.9. Real-Time Outlier Detection and Flow Forecasting

To emulate the application of this work in a time-of-purchase distribution system, a flowchart was developed to illustrate the integrated operation of the system. In this scenario, an online environment is created for the simultaneous application of TEDA and ANN.

Using IoT technology, pressure and flow data are continuously monitored and transmitted to a broker hosted on AWS. SCADA LTS plays a crucial role as it reads these measurements in real time and, when it detects changes, sends commands to the inverter responsible for pressurizing the laboratory’s distribution network.

The variation in pressure and flow measurements at the measuring points makes it possible for the IoT system to acquire this information accurately. When the data is sent to the broker, a verification process is carried out to identify possible outliers. If atypical data is detected, it is automatically replaced by the last recorded reading, ensuring that the system maintains data integrity.

The ANN, in turn, is constantly monitoring, analyzing the new values available and making predictions for the two future flows. These forecasts are stored in the broker and can be viewed graphically in SCADA LTS, providing valuable visual feedback on the system’s performance.

**Figure 28:**
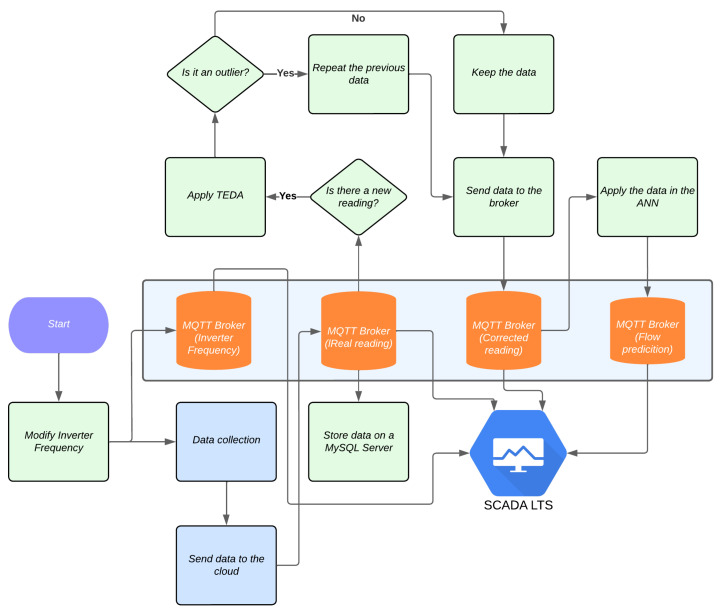
Experimental flowchart of TEDA and RNA running in real time. Source: authors.

Figure 28 illustrates the flowchart of this scenario implemented in the laboratory, detailing the interaction between the system’s components. The application of TEDA to detect outliers, combined with the predictive capacity of the ANN, not only increases the accuracy of the readings but also allows dynamic adjustments in real time, adapting the system to changing conditions and ensuring more reliable operation.

These tools and processes highlight the importance of integrating IoT technologies, anomaly detection algorithms, and neural networks for efficient real-time monitoring of distribution systems.

**Figure 29:**
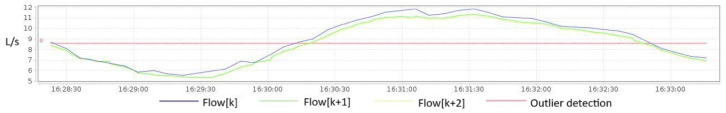
Real-time online flow rate prediction using ANN and TEDA. Source: authors.

Using SCADA LTS, it is possible to observe the behavior of the system in real time, as shown in Figure 29. The flow curves are evident, with the blue curve being the measured flow and the green curve being the forecast using the proposed methodology. In addition, the TEDA algorithm checks for outliers.

## 9. Conclusions and Future Works

In summary, this work presented the development of an energy self-sufficient IoT monitoring node, powered by a micro-hydraulic turbine, capable of operating continuously in water supply networks without the need for external energy sources. The proposed solution incorporated the integration of the TEDA algorithm with an ANN, enabling real-time outlier detection and correction, and surpassing the performance of conventional filtering methods. Additionally, the use of an ANN as a soft sensor enabled predictions with a mean absolute percentage error (MAPE) close to 3%, highlighting the robustness of the model. The experiments carried out under controlled laboratory conditions confirmed the technical feasibility and energy efficiency of the system, demonstrating its potential for real-world applications, particularly in reducing maintenance and operational costs, while also contributing to water efficiency and sustainability in urban distribution systems.

This research presented an IoT-based solution, which can be energetically autonomous, cost-effective, energy-efficient, and capable of remotely monitoring pressure and flow in a water supply network. Using advanced technologies, including wireless sensors and low-power-consumption techniques, this system collects and transmits data, simplifying the monitoring and management of water distribution.

This research illustrates how IoT applications can offer practical alternatives to networks of traditional sensors, especially in large-scale urban environments where cost and viability are important considerations. The proposed system can be part of a solution to overcome recurring challenges in water distribution systems.

The results showed that the application of TEDA enabled outliers to be identified and corrected, improving the quality of the data used by the ANN for its predictions. When the ANN was trained without the presence of outliers, it was possible to obtain an MAPE of 3.15%, showing an accurate forecast of consumption demand. However, by introducing outliers only in the test data, the MAPE increased to 5.16%, indicating a reduction in forecast accuracy. On the other hand, by applying TEDA to detect outliers, the ANN was able to improve its performance, resulting in an MAPE of 3.04%, demonstrating the effectiveness of outlier detection.

In the current scenario, where losses in distribution systems are significantly high, the implementation of these technologies becomes even more crucial. As presented in the introductory chapter, losses can occur due to leaks, sensor reading failures, or unexpected variations in measurements. TEDA’s ability to detect and mitigate the influence of outliers, combined with ANN’s flow prediction, offers a solution for improving the management and efficiency of water distribution systems. This approach not only reduces financial losses but also contributes to more sustainable use of increasingly scarce water resources.

Future work will focus on improving and expanding this solution, aiming for a more efficient, sustainable, and resilient water supply system. Based on the results achieved in this research, a range of applications are revealed, such as the simulation of leaks in the distribution system, generating alerts for the operator based on the error presented by the difference between the predicted and measured flow. In this context, the use of TEDA with leakage data can be investigated.

## Figures and Tables

**Table 1:** Project costs. Source: authors.

Items	Quantity	Unitary Value	Cost
PCB (Board and Components)	-	-	USD 4.11
ESP32 Module (38 pins)	1	USD 16.44	USD 16.44
LM2596 Module	1	USD 4.11	USD 4.11
Microturbine	1	USD 8.24	USD 8.24
Battery	1	USD 23.40	USD 23.40
Encapsulation	1	USD 10.27	USD 10.27
Other Costs	-	-	USD 4.11

**Table 2:** Table of interpolated outliers. Source: authors.

Index	Actual Value	Interpolated Value	Relative Error
13	12.30	12.82	USD 4.26
17	14.55	USD 15.07	USD 3.60
30	21.30	USD 21.37	USD 0.35
36	21.75	USD 21.30	USD 2.06
39	20.55	USD 20.70	USD 0.72

## Data Availability

No new data were created or analyzed in this study. Data sharing is not applicable to this article.

## References

[1] WHO, UNICEF (2000). Global Water Supply and Sanitation Assessment 2000 Report.

[2] Gheisi A., Forsyth M., Naser G. (2016). Water Distribution Systems reliability: A review of Research Literature. J. Water Resour. Plan. Manag..

[3] Rossman L., Woo H., Tryby M., Shang F., Janke R., Haxton T. (2021). EPANET 2.2 User Manual.

[4] Bairrogi A.G., Parad R., Baeza V.M., Monzo C. (2024). Leveraging Urban Water Distribution Systems with Smart Sensors for Sustainable Cities. Sensors.

[5] Åström K., Murray R. (2021). Feedback Systems: An Introduction for Scientists and Engineers.

[6] Ueyama J., Hughes D., Man K.L., Guan S.U., Matthys N., Horré W., Michiels S., Huygens C., Wouter J. (2010). Applying a multiparadigm approach to implementing wireless sensor network based river monitoring. Proceedings of the First ACIS International Symposium on Cryptography and Network Security, Data Mining and Knowledge Discovery, E-Commerce Its Applications and Embedded Systems (CDEE).

[7] Hassam Q.F., Madani S.A., Khan A.u.R. (2017). Internet of Things: Challenges, Advances, and Applications.

[8] Osama M., Ateya A.A., Sayed M.S., Hammad M., Plawiak P., El-Latif A.A.A., Elsayed R.A. (2023). Internet of Medical Things and Healthcare 4.0: Trends, Requirements, Challenges, and Research Directions. Sensors.

[9] Furquim G., Pessin G., Faiçal B.S., Mediondo E.M., Ueyama J. (2016). Improving the accuracy of a flood forecasting model by means of machine learning and chaos theory. Nat. Comput. Appl..

[10] Kim S., Khan I.C.S.K.Y. (2021). Earthquake Alert Device Using a Low-Cost Accelerometer and its Services. IEEE Access.

[11] Aljohani F., Sen A., Ramazan M., Alzahrani B., Bahbouh N. (2023). A Smart Framework for Managing Natural Disasters Based on the IoT and ML. Appl. Sci..

[12] Zeng F., Chuan P., Tang H. (2023). Sensors on the Internet of Things Systems for Urban Disaster Management: A Systematic Literature Review. Sensors.

[13] Santoshi R., Rughani P. (2023). IoT based Agriculture (Ag-IoT): A detailed study on Architecture, Security and Forensics. Inf. Process. Agric..

[14] Yan Z., Gang H. (2019). Design of Intelligent Water Metering System for Agricultural Water Based on NB-IOT. Proceedings of the 2019 IEEE 3rd Advanced Information Management, Communicates, Electronic and Automation Control Conference (IMCEC 2019).

[15] Aslam M.M., Tufail A., Kim K.H., Apong R.A.A.H.M., Raza M.T. (2023). A Comprehensive Study on Cyber Attacks in Communication Networks in Water Purification and Distribution Plants: Challenges, Vulnerabilities, and Future Prospects. Sensors.

[16] Baek S.H., Lee K.I., Kim S.M. (2023). Development of Real-Time Monitoring System Based on IoT Technology for Curing Compound Application Process during Cement Concrete Pavement Construction. Sensors.

[17] Alharbi H.A., Aldossary M. (2021). Energy-Efficient Edge-Fog-Cloud Architecture for IoT-Based Smart Agriculture Environment. IEEE Access.

[18] Mohapatra H., Rath A.K. (2022). IoE based framework for smart agriculture. J. Ambient. Intell. Humaniz. Comput..

[19] Wang Q.M., Abdelrahman W. (2023). High-Precision AI-Enabled Flood Prediction Integrating Local Sensor Data and 3rd Party Weather Forecast. Sensors.

[20] Sangiorgi L., Sberveglieri V., Carnevale C., De Nardi S., Nunez-Carmona E., Raccagni S. (2024). Data-Driven Virtual Sensing for Electrochemical Sensors. Sensors.

[21] Morais Júnior A.A.d., Brito R.P., Sodré C.H. (2014). Design of a Soft Sensor with Technique Neuro-Fuzzy to Infer the Product Composition a Distillation Process. Lect. Notes Eng. Comput. Sci..

[22] He Y., Wang P., Zhu Q. (2023). An Improved Industrial Process Soft Sensor Method Based on LSTM. Proceedings of the 2023 IEEE 12th Data Driven Control and Learning Systems Conference (DDCLS).

[23] Lima J.d.S., Villanueva J.M.M., Catunda S.Y.C. (2022). Modeling a Virtual Flow Sensor in a Sugar-Energy Plant using Artificial Neural Network. Proceedings of the 2022 IEEE International Instrumentation and Measurement Technology Conference (I2MTC).

[24] Shan B., Ma C., Niu C., Xu Q., Zhu Z., Wang Y., Zhang F. (2023). Soft sensor model predictive control for azeotropic distillation of the separation of DIPE/IPA/water mixture. J. Taiwan Inst. Chem. Eng..

[25] Xu B., Pooi C.K., Tan K.M., Huang S., Shi X., Ng H.Y. (2023). A novel long short-term memory artificial neural network (LSTM)-based soft-sensor to monitor and forecast wastewater treatment performance. J. Water Process Eng..

[26] Lima R.P.G., Villanueva J.M.M., Gomes H.P., Flores T.K.S. (2022). Development of a Soft Sensor for Flow Estimation in Water Supply Systems Using Artificial Neural Networks. Sensors.

[27] Alencar G.M.R.d., Fernandes F.M.L., Duarte R.M., Melo P.F.d., Cardoso A.A., Gomes H.P., Villanueva J.M.M. (2024). A Soft Sensor for Flow Estimation and Uncertainty Analysis Based on Artificial Intelligence: A Case Study of Water Supply Systems. Automation.

[28] Jewel M.H., Mamun A.A. (2022). Internet of Things (IoT) for Water Quality Monitoring and Consumption Management. Proceedings of the 2022 4th International Conference on Sustainable Technologies for Industry 4.0 (STI).

[29] Giuliano F., Pagano A., Croce D., Vitale G., Tinnirello I. (2023). Adaptive Algorithms for Batteryless LoRa-Based Sensors. Sensors.

[30] Chowdhury A.A.S., Arafat Y.A., Alam M.S. (2022). IoT-GSM Based Controlling and Monitoring System to Prevent Water Wastage, Water Leakage, and Pollution in the Water Supply. Proceedings of the 2022 International Conference on Innovations in Science, Engineering and Technology (ICISET).

[31] Paulraj G.J.L., Jebadurai I.J., Jebadurai J. (2022). IoT-Based Smart Water Tank Supply Management System Using MQTT Protocol. Proceedings of the 2022 Seventh International Conference on Parallel, Distributed and Grid Computing (PDGC).

[32] Subha J., Kowsigan M. (2022). Optimal Water Supply Scheduling Mechanisms using Bio-Inspired Algorithms for IoT-Based Smart Water Distribution Network. Proceedings of the International Conference on Augmented Intelligence and Sustainable Systems (ICAISS-2022).

[33] Castellano J.M.R., Cabrera J.M.D., González I.L.C., Baena A.J.G., Ortega A.M.R., Miranda A.L.M. (2023). Solución iot para la gestión eficiente de las redes de abastecimiento de agua. Proceedings of the XVII Congreso Iberoamericano sobre Sistemas de Abastecimiento, Saneamiento y Riego (SEREA23).

[34] Ebisi F., Nikolakakos I.P., Karunamurthi J.V., Binahmed A.A.N.A., Al B.E., Alblooshi S. (2023). Machine learning schemes for leak detection in IoT-enabled water transmission system. Proceedings of the 2023 International Conference on IT Innovation and Knowledge Discovery (ITIKD).

[35] Saritha G., Ishwarya R., Saravanan T., Sudarshana P.A.S., Sowmiya S. (2023). Water Quality Monitoring System Using IoT. Proceedings of the 2023 Eighth International Conference on Science Technology Engineering and Mathematics (ICONSTEM).

[36] Chowdhury A.A.S., Arafat Y.A., Alam M.S. (2023). Indigenous Development of Water Quality Monitoring System for Urban Areas using IoT & ML. Proceedings of the 2023 International Conference on Computer, Electronics Electrical Engineering their Applications (IC2E3).

[37] Boebel M., Frei F., Blumensaat F., Ebi C., Meli M.L., Rüst A. (2023). Batteryless Sensor Devices for Underground Infrastructure—A Long-Term Experiment on Urban Water Pipes. J. Low Power Electron. Appl..

[38] Syrmos E., Sidiropoulos V., Bechtsis D., Stergiopoulos F., Aivazidou E., Vrakas D., Vezinias P., Vlahavas I. (2023). An Intelligent Modular Water Monitoring IoT System for Real-Time Quantitative and Qualitative Measurements. Sustainability.

[39] Puviyarasi B., Gubhenthiran P., Keerthana P., Megala S. (2019). Water quality monitoring and cleaning system. Proceedings of the International Conference on Communication, Computing and Internet of Things (IC3IoT).

[40] Aragonés R., Oliver J., Ferrer C. (2025). Enhanced Heat-Powered Batteryless IIoT Architecture with NB-IoT for Predictive Maintenance in the Oil and Gas Industry. Sensors.

[41] Bansal R.C. (2005). Three-phase self-excited induction generators: An overview. IEEE Trans. Eneergy Convers..

[42] Madakam S., Ramaswamy R., Tripathi S. (2015). Internet of things (IoT): A literature review. J. Comput. Commun..

[43] Gatial E., Balogh Z., Hluchỳ L. (2022). Concept of energy efficient ESP32 chip for industrial wireless sensor network. Proceedings of the 2020 IEEE 24th International Conference on Intelligent Engineering Systems (INES).

[44] Peterson T.J., Western A.W.C.X. (2018). The good, the bad and the outliers: Automated detection of errors and outliers from groundwater hydrographs. Hydrogeol. J..

[45] Figueiredo A.A.d.O., Cabral J.J.d.S.P., Silva S.R.d., Bezerra S.d.T.M. (2023). Avaliação e potencial de redução de perdas de água em cidades do estado de pernambuco com escassez hídrica e abastecimento intermitente. J. Environ. Anal. Prog. (JEAP).

[46] Rousseeuw P.J., Croux C. (1993). Alternatives to the Median Absolute Deviation. J. Am. Stat. Assoc..

[47] Angelov P.P. (2014). Outside the box: An alternative data analytics framework. J. Autom. Mob. Robot. Intell. Syst..

[48] Costa B.S.J., Bezerra C.G.B., Guedes L.A., Angelov P.P. (2015). Online fault detection based on Typicality and Eccentricity Data Analytics. Proceedings of the 2015 International Joint Conference on Neural Networks (IJCNN).

[49] Bernieri A., Betta G.F.D., Liguori C. (1996). On-line fault detection and diagnosis obtained by implementing neural algorithms on a digital signal processor. IEEE Trans. Instrum. Meas..

[50] Burden R.L., Faires J.D. (2015). Numerical Analysis.

[51] Kriegeskorte N., Golan T. (2019). Neural network models and deep learning. Curr. Biol..

[52] Chen L., Li S., Bai Q., Yang J., Jiang S., Miao Y. (2021). Review of Image Classification Algorithms Based on Convolutional Neural Networks. Remote Sens..

[53] Kingma D.P., Ba J. Adam: A Method for Stochastic Optimization. Proceedings of the 3rd International Conference for Learning Representations (ICLR’15).

[54] Cominato C., Sborz J., Kalbusch A., Henning E. (2022). Water demand profile before and during COVID-19 pandemic in a Brazilian social housing complex. Heliyon.

[55] Angelov P. (2012). Autonomous Learning Systems: From Data Streams to Knowledge in Real-Time.

